# NLRP3 Inflammasome Is Expressed and Functional in Mouse Brain Microglia but Not in Astrocytes

**DOI:** 10.1371/journal.pone.0130624

**Published:** 2015-06-19

**Authors:** Audrey Gustin, Mélanie Kirchmeyer, Eric Koncina, Paul Felten, Sophie Losciuto, Tony Heurtaux, Aubry Tardivel, Paul Heuschling, Catherine Dostert

**Affiliations:** 1 Life Sciences Research Unit, Faculty of Science, Technology and Communication, University of Luxembourg, Luxembourg, Luxembourg; 2 Biochemistry Institute, University of Lausanne, Epalinges, Switzerland; University Hohenheim, GERMANY

## Abstract

Neuroinflammation is the local reaction of the brain to infection, trauma, toxic molecules or protein aggregates. The brain resident macrophages, microglia, are able to trigger an appropriate response involving secretion of cytokines and chemokines, resulting in the activation of astrocytes and recruitment of peripheral immune cells. IL-1β plays an important role in this response; yet its production and mode of action in the brain are not fully understood and its precise implication in neurodegenerative diseases needs further characterization. Our results indicate that the capacity to form a functional NLRP3 inflammasome and secretion of IL-1β is limited to the microglial compartment in the mouse brain. We were not able to observe IL-1β secretion from astrocytes, nor do they express all NLRP3 inflammasome components. Microglia were able to produce IL-1β in response to different classical inflammasome activators, such as ATP, Nigericin or Alum. Similarly, microglia secreted IL-18 and IL-1α, two other inflammasome-linked pro-inflammatory factors. Cell stimulation with α-synuclein, a neurodegenerative disease-related peptide, did not result in the release of active IL-1β by microglia, despite a weak pro-inflammatory effect. Amyloid-β peptides were able to activate the NLRP3 inflammasome in microglia and IL-1β secretion occurred in a P2X7 receptor-independent manner. Thus microglia-dependent inflammasome activation can play an important role in the brain and especially in neuroinflammatory conditions.

## Introduction

Inflammatory processes have been implicated in both acute and chronic neurodegenerative conditions. Pro-inflammatory cytokine production has been associated with neuroinflammation and different neurodegenerative diseases, such as Alzheimer’s disease (AD), Parkinson’s disease (PD) and Multiple Sclerosis (MS) [1–3]. Although inflammation may not typically represent an initiating factor in neurodegenerative diseases, there is emerging evidence in animal models that sustained inflammatory responses involving microglia and astrocytes contribute to disease progression [4]. Among these responses, IL-1β secretion has been shown to have important functions in diseases of the Central Nervous System (CNS), yet the precise mechanism of action is not clear since both neurotoxic and regenerative roles have been described [5–7].

The molecular steps leading to IL-1β maturation take place in an intracellular complex termed the inflammasome [8]. The inflammasome is a caspase-1 activating multiprotein platform that results from oligomerization of inactive monomeric proteins from the nucleotide-binding domain, leucine-rich repeat (NLR) protein family. Different complexes have been described and are usually defined by the core NLR proteins that are implicated, such as NLRP1, NLRP3, NLRC4 (also called IPAF) or the HIN200 family member AIM2. The most intensively studied is the NLRP3 inflammasome which is formed when NLRP3 associates with the adaptor protein ASC and procaspase-1 upon activation by different stimuli, such as pathogen associated molecular patterns (PAMPs) or endogenous danger signals [9, 10]. Inflammasome assembly results in the cleavage of caspase-1 from its proform to its enzymatically active form. This active caspase-1 then processes the cleavage of several substrates, such as pro-IL-1β and pro-IL-18 into the mature cytokines IL-1β and IL-18. IL-1α, another IL-1 family member, and High-mobility group box 1 (HMGB1) do not require cleavage by caspase-1 but their release in response to certain stimuli is dependent on the inflammasome [11, 12]. All these cytokines are shown to be important actors in different neurodegenerative diseases [13, 14]. The precise mechanism of inflammasome activation is not fully understood. NLRP3 can sense a variety of molecular structures and it is thought that inflammasome activation occurs probably not through direct ligand-receptor interaction but rather the sensing of cellular homeostasis disruption [15, 16].

The role of the NLRP3 inflammasome in CNS diseases has recently started to be investigated in more detail. Several studies suggest a general involvement of the NLRP3 inflammasome in MS, a demyelinating inflammatory disease of the CNS. In experimental autoimmune encephalomyelitis (EAE), a mouse model of MS, inflammasome-deficient mice are protected from disease progression (reviewed in [17]). In addition to MS, the implication of the NLRP3 inflammasome in Alzheimer's disease has been shown both *in vitro* by the ability of amyloid-beta (Aβ) peptides to activate the inflammasome [18] and *in vivo* in an AD mouse model where NLRP3 knock-out mice were protected from spatial memory impairment and showed decreased Aβ plaque burden [19].

In many cases, neurodegenerative diseases are related to an accumulation of protein aggregates such as Lewy bodies in PD, senile plaques in AD or PrP^sc^ in prion related-disease. As shown in a recent study, PrP fibrils are able to induce inflammasome-dependent IL-1β secretion in microglia, suggesting a role for the NLRP3 inflammasome in prion diseases [20, 21]. Besides, the capacity of similar aggregates, such as islet amyloid polypeptide (IAPP) in type II diabetes, to activate NLRP3 inflammasome via abnormal phagocytosis and lysosomal rupture mechanism has also been shown [22].

In view to understand the role played by IL-1β in neurodegenerative diseases, it is important to characterize in detail the inflammasome expression and activation in CNS inflammatory cells. Microglia are the resident macrophages of the brain and as such are described to secrete IL-1β. Astrocytes are the most abundant cell type in the brain where they maintain CNS homeostasis and provide neuronal support in healthy conditions. In the injured brain they play important roles during glial scar formation, a process referred to as reactive astrogliosis [23]. However it is not entirely clear if astrocytes are able to produce IL-1β. In addition there are only few studies about the role astrocytes can play during neuroinflammation with regard to inflammasome activation [24]. Therefore we aimed to investigate the relative contribution of microglia and astrocytes to inflammasome activation in the context of neurodegenerative disease-related peptide stimulation.

## Materials and Methods

### Mice

C57BL/6JOlaHsd mice were from Harlan. *Nlrp3* ^*-/-*^ mice were provided by University of Lausanne [25]. *Casp1* ^*-/-*^ */Casp11* ^*-/-*^ (sn#016621) (hereafter referred to as *Casp1* ^*-/-*^) and *P2rx7* ^*-/-*^ (sn#005576) mice were obtained from The Jackson Laboratory (Bar Harbor, USA). All mouse strains were housed and bred at the animal-housing facility of the University of Luxembourg approved by the National Veterinary Inspection. All animal procedures were conducted in strict accordance with the FELASA Guide for the Care and Use of Laboratory Animals. The protocol was approved by the animal ethics and experimentation committee (AEEC) of the University of Luxembourg. All efforts were made to minimize suffering. Newborn mice were decapitated.

### Cell cultures

Mixed glial cell cultures were prepared as previously described [26]. Briefly, after carefully removing meninges and large blood vessels, the brains from newborn mice were pooled and then minced in cold phosphate-buffered saline (PBS) solution. After mechanical dissociation cell suspensions were washed and plated in complete medium (Dulbecco’s Modified Eagle Medium (DMEM, Sigma-Aldrich) supplemented with 10% fetal bovine serum (FBS, Gibco), 100 U/ml penicillin and 100 U/ml streptomycin, both from Lonza) at 37°C in a humidified atmosphere containing 5% CO_2_. The culture medium was changed after three days of culture. After 15 days, mixed glial cultures had reached confluence. Glial cells were separated by magnetic-activated cell sorting (MACS), as previously described [26]. Microglia were positively selected using an anti-CD11b antibody, following the manufacturer’s instructions (Miltenyi), and plated in mixed glial culture conditioned medium to be treated twenty-four hours later. Astrocytes were negatively sorted during this process. The obtained “astrocyte-enriched cultures” were plated in 75 cm^2^ flasks for seven days allowing the cultures to reach confluence. After one week, the MACS procedure was repeated in order to reduce microglial contamination of the astrocyte population.

Neurosphere-derived astrocytes were prepared as described in [27]. Briefly, neurospheres derived from embryonic neural stem cells were cultured and were differentiated to astrocytes by plating them on poly-L-ornithine coated flasks in complete DMEM.

Bone-marrow derived macrophages (BMDM) were generated as previously described [28].

Cortical neuron cultures were prepared from E15 mouse embryos as previously described [29].

### In vitro stimulation experiments

Cells were primed with ultrapure LPS (10 ng/ml), P3C (100 ng/ml) (both from Invivogen), IL-1β(10 ng/ml), TNFα(10 ng/ml), IFNγ (20 ng/ml) or a Complete Cytokine Mix (CCM: 10 ng/ml IL-1β, 10 ng/ml TNFα and 20 ng/ml IFNγ) (all from R&D). Stimulation with inflammasome activators (ATP, Nigericin (both from Sigma-Aldrich), MSU (Invivogen), Alum (Pierce), poly(dA:dT) (Invivogen)) was performed as indicated in the figure legends. The amyloid beta peptide 25–35 and the reverse form 35–25 (Sigma-Aldrich) were dissolved in PBS and aliquoted at −20°C. The amyloid beta peptide 1–42 (Bachem) was used under oligomeric and fibrillar form. Fibrillar form was obtained by incubation at 37°C during 7 days in DMEM. WT and A53T mutant α-synuclein were purchased from rPeptide. Aliquots were resuspended in H_2_O to obtain a 100 μM solution. The preparation was incubated for 4 days at 54°C with shaking to obtain fibrillar α-synuclein or was used directly for oligomeric α-synuclein activation. Where indicated in the legends, cells were treated with high KCl (Sigma-Aldrich) medium or inhibitors (N-acetyl cystein (NAC) 5 mM (Sigma-Aldrich), (L- 3- trans- (Propylcarbamoyl)oxirane- 2- Carbonyl)- L- Isoleucyl- L- Proline Methyl Ester (Ca-074Me) 10 μM (PeptaNova), or cytochalasin D (cytoD) 2 μM (Sigma-Aldrich)) 30 min prior to addition of ATP or Alum.

### Real-time PCR

Total RNA was isolated using the Invisorb Spin Cell RNA Mini Kit (Invitek) according to the manufacturer’s protocol. Complementary DNA (cDNA) was synthesized from RNA samples using the ImProm-II Reverse Transcription System (Promega). Gene expression was analyzed using Bio-Rad iCycler (iQ5 Real-Time PCR Detection System, Bio-Rad Laboratories) with SYBR Green Supermix (Promega). Expression was normalized relative to the housekeeping gene L27. Primer sequences were designed using the Beacon Designer Software (Bio-Rad) (Table 1).

**Table 1:** Primer sequences list

Gene	Accession number	Primers sequence
***Aif1***	NM_019467	dir : TTCCCAAGACCCACCTAG
		rev : TCCTCATACATCAGAATCATTC
***Aim2***	NM_001013779	dir : ATAGGAGGAACAACAACAT
		rev : GCCATCTTCTGCTACATA
***Asc***	NM_011977	dir : AGGAGTGGAGGGGAAAGC
		rev : AGAAGACGCAGGAAGATGG
***Casp1***	NM_009807	dir : AGGAATTCTGGAGCTTCAATCAG
		rev : TGGAAATGTGCCATCTTCTTT
***Casp4***	NM_007609	dir : GCTCTTACTTCATCACTA
		rev : AATATCTCGTCAAGGTTG
***Cxcl10***	NM_021274	dir: TTCTGCCTCATCCTGCTG
		rev: AGACATCTCTGCTCATCATTC
***Gfap***	NM_010277	dir : GGTTGAATCGCTGGAGGAG
		rev : CTGTGAGGTCTGGCTTGG
***Hmgb1***	NM_010439	dir : TGGCAAAGGCTGACAAGGCTC
		rev : GGATGCTCGCCTTTGATTTTGG
***Il18***	NM_008360	dir : ACCAAGTTCTCTTCGTTGAC
		rev : TCACAGCCAGTCCTCTTAC
***IL1a***	NM_10554	dir : AAGACAAGCCTGTGTTGCTGAAGG
		rev : TCCCAGAAGAAAATGAGGTCGGTC
***IL1b***	NM_008361	dir : GCTTCAGGCAGGCAGTATC
		rev : AGGATGGGCTCTTCTTCAAAG
***Il33***	NM_133775	dir : CAATGACCAATCTGTTAGT
		rev : CATAGTAGCGTAGTAGCA
***Il6***	NM_031168	dir : ACCGCTATGAAGTTCCTCTC
		rev : CTCTGTGAAGTCTCCTCTCC
***Itgam***	NM_008401	dir : TGGACGCTGATGGCAATACC
		rev: GGCAAGGGACACACTGACAC
***Nlrc4***	NM_001033367	dir : GTCAAGTGTTATCCAAGTTA
		rev : CGCTAATATCATAGTCATCAA
***Nlrp1***	NM_001004142	dir : GGTGTGCTGGTTGGTCTGC
		rev : GTGCTGTGGTGGTCTGTGAG
***Nlrp12***	NM_001033431	dir : AAGAGATGAGATGCTACCTTGAGAG
		rev : ATGCCAACACTTCCTCCTTCAC
***Nlrp2***	NM_177690	dir : AAGCCTGTAGAGGTCTTACTG
		rev : ACTGTGTCCGTGTGGTTAC
***Nlrp3***	NM_145827	dir : GCTCCAACCATTCTCTGACC
		rev : AAGTAAGGCCGGAATTCACC
***Nlrp6***	NM_001081389	dir : GGACGAGAGGAAGGCAGAG
		rev : GCACACGAAGGGCACAAAG
***Nos2***	NM_011198	dir : AGCCCTCACCTACTTCCTG
		rev : CAATCTCTGCCTATCCGTCTC
***P2x7r***	AJ_009823	dir : GGACCCACAGAGCAAAGGAATC
		rev : TCAGTAGGACACCAGGCAGAG
***Ptgs2***	NM_010927	dir : GCCTGGTCTGATGATGTATGC
		rev : GAGTATGAGTCTGCTGGTTTGG
***Rpl27***	NM_011289	dir : ACATTGACGATGGCACCTC
		rev : GCTTGGCGATCTTCTTCTTG
***Tlr4***	NM_021297	dir : TTCACCTCTGCCTTCACTAC
		rev : CACTACCACAATAACCTTCCG
***Tnf***	NM_013693	dir : GGTTCTGTCCCTTTCACTCAC
		rev : TGCCTCTTCTGCCAGTTCC

### Immunoblot analysis

Cell extracts and precipitated supernatants were analyzed by immunoblot using goat anti-IL-1β (R&D), mouse anti-NLRP3 (Cryo-2), rabbit anti-ASC (AL177), mouse anti-Caspase-1 (Casper-1) (all three from Adipogen), and mouse anti-alpha-tubulin (Abcam).

### Protein quantification assays

Cell culture supernatants were assayed, at the indicated times, for the presence of cytokines by using mouse IL-1β, TNFα, CXCL10 DuoSet ELISA and IL-1α DuoSet ELISA (all R&D) or IL-18 ELISA (MBL) according to the manufacturer’s instructions. ATP was quantified in cell supernatants using ATP Determination Kit (Life technologies).

### Immunocytochemistry

Cells were cultured on poly-L-lysine coated coverslips and fixed with paraformaldehyde (4% in PBS) after stimulation. Cells were incubated overnight at 4°C with Cy3-labeled mouse anti-GFAP (1:800, Sigma-Aldrich), rabbit anti-Iba1 (1:200, Biocare Medical), goat anti-IL-1β (1:100, R&D), mouse anti-NLRP3 (1:100), rabbit anti-ASC (1:200), mouse anti-Caspase-1 (1:300, all three Adipogen) and rabbit anti-HMGB1 (1:400, Abcam). After washing with PBS, cells were incubated with anti-rabbit, anti-goat or anti-mouse secondary antibodies coupled to either Cy3 or AlexaFluor 488 (Invitrogen). Cells were then washed with PBS and mounted with DAPI-Fluoromount G (SouthernBiotech, USA), and observed under a LSM 510 META inverted confocal microscope (Carl Zeiss Micro Imaging, Göttingen, Germany).

### Statistical analysis

The significance of multiple treatments was analyzed by a one way analysis of variance (anova) followed by Tukey’s or Dunnett’s multiple comparisons tests. If multiple treatments were tested on different genotypes, a two-way anova was performed. The homoscedasticity and normality assumptions were assessed respectively by the Bartlett and Shapiro-Wilk tests. The statistical analysis of gene expression was performed on the delta-Ct values (Ct_goi_-Ct_hkg_) and the analysis of protein quantification (Elisa) was performed on log-transformed concentrations (if present, zero values were removed by adding an offset to the dataset equal to half the smallest non-zero concentration). Non normal data was analyzed by a non-parametric analysis of variance (Kruskal–Wallis test) followed by pairwise comparisons with Mann–Whitney U-tests and Holm’s correction.

All statistical analyses were performed using R (http://www.r-project.org/) and differences with p-values less than 0.05 were considered significant. All experiments have been performed at least three times.

## Results

### Brain microglia, but not astrocytes, express NLRP3 inflammasome components and substrates

**Fig 1:**
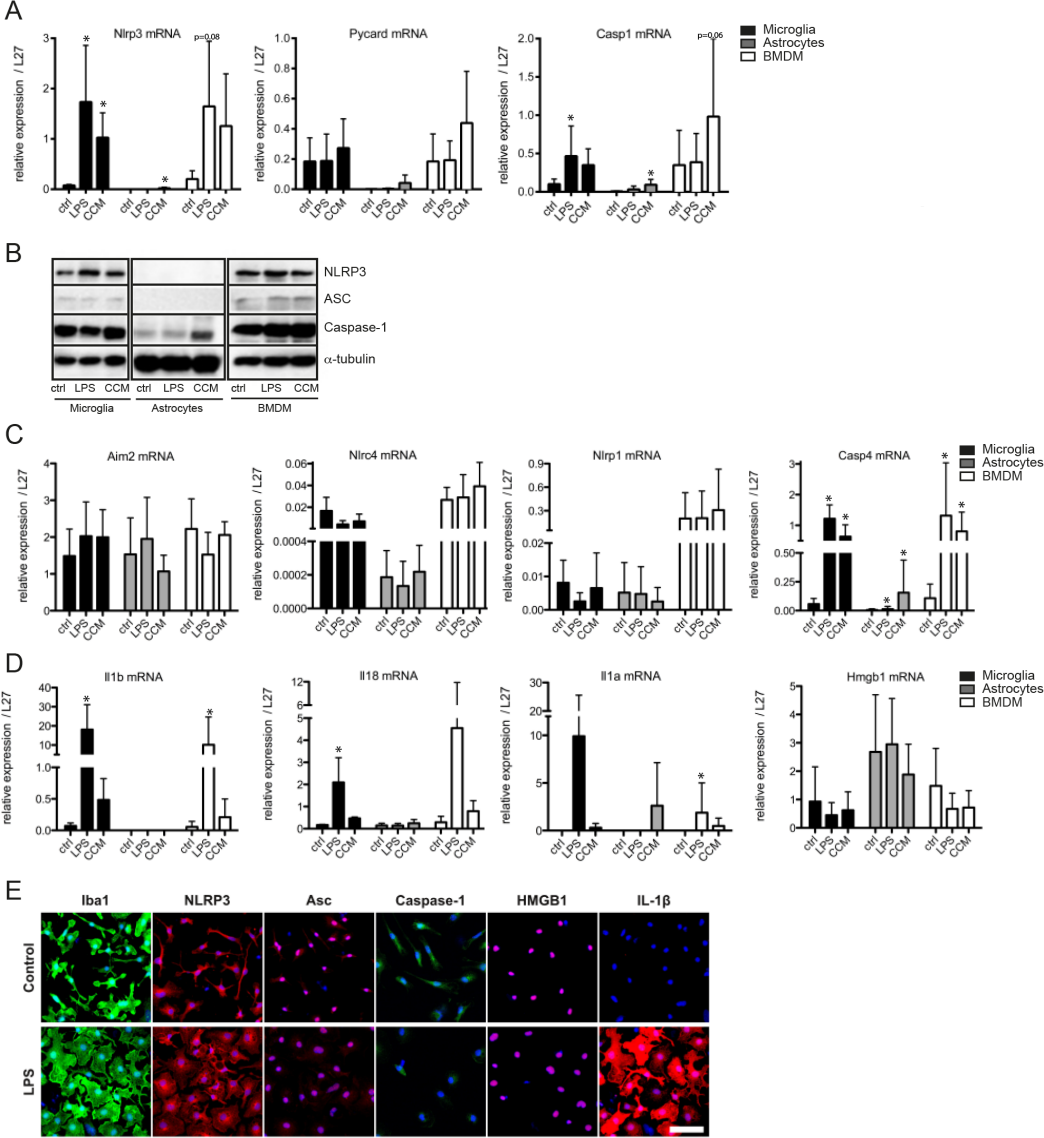
Inflammasome components NLRP3, ASC and caspase-1 are expressed in microglia, but not in astrocytes. mRNA and/or protein expression of inflammasome components and substrates NLRP1, NLRP3, NLRC4, ASC (Pycard), Aim-2, Caspase-1, Caspase-4, IL-1α, IL-1β, IL-18 and HMGB1, were studied in mouse microglia, astrocytes, or bone-marrow derived macrophages (BMDM) after 6 hours of stimulation with LPS or Complete Cytokine Mix. RNA was extracted and analyzed for gene expression, relative to *L27*, by Real-Time PCR (**A, C, D**). Bar graphs shown represent the mean ± SD. (**B**) NLRP3, ASC and Caspase-1 expression were analyzed by Western Blot and α-Tubulin was used as loading control. (**E**) Microglia were analyzed by immunofluorescence for the expression of Iba1, NLRP3, ASC, Caspase-1, HMGB1 and IL-1β, nuclei are stained by DAPI, scale bar: 50μm. Data shown are the mean ± SD of at least three independent experiments. *p<0.05 compared to control (ctrl).

In order to better understand inflammasome function in the brain, especially in the glial compartment, we aimed to analyze NLRP3, ASC and caspase-1 expression in the different cell types. Expression of inflammasome components was monitored at both the transcript and the protein level (Fig 1A and 1B). Microglia express inflammasome components NLRP3, ASC (*Pycard*) and caspase-1, similarly to bone marrow-derived macrophages that were used as control cells (Fig 1A and 1B). ASC and caspase-1 were constitutively present in microglia, whereas NLRP3 expression could be upregulated after priming with LPS. In addition to priming with LPS we used a complete cytokine mix (CCM: IL-1β, TNFα and IFNγ) that was also able to induce NLRP3 expression in microglia and BMDMs (Fig 1A and 1B).

In astrocytes however, although caspase-1 was expressed at low levels, only very weak levels of NLRP3 and ASC transcript and almost no NLRP3 and ASC protein could be detected, suggesting that these cells probably lack a functional NLRP3 inflammasome (Fig 1A and 1B). CCM was used in order to prime astrocytes as they do only weakly express TLR4 (Figure A in S1 File) and do not strongly respond to LPS stimulation [30]. However, we were still unable to robustly prime the inflammasome in astrocytes, as shown by the very moderate upregulation of NLRP3. Also priming of astrocytes with several other agents (e.g. P3C, as shown below), as well as combinations thereof (e.g. CCM and P3C, data not shown), did not result in significant expression of NLRP3 and/or IL-1β.

We further investigated expression of other inflammasome related proteins and found that both microglia and astrocytes expressed similar transcript levels of the intracellular sensors *Aim2* and *Nlrp1*, whereas *Nlrc4* was mainly expressed in microglia (Fig 1C). Interestingly, *Casp4* (caspase-11) mRNA could be upregulated in microglia and astrocytes after priming (Fig 1C and Figure C in S1 File). In addition, classical inflammasome substrates, such as *Il1b* and *Il18*, were strongly induced by LPS priming in microglia. *Il1b* transcript remained virtually undetectable in astrocytes after CCM priming and *Il18* basal expression levels remained unchanged after priming (Fig 1D). *Il1a* expression was inducible by LPS treatment in microglia and by CCM treatment in astrocytes. *Hmgb1* was expressed by both microglia and astrocytes at mRNA and protein level (Fig 1D and 1E). Protein localization was monitored by immunocytochemistry in microglia, where LPS priming resulted in morphological changes monitoring cell activation, shown by both cell surface marker Iba1 and IL-1β expression (Fig 1E). The inflammasome components NLRP3, ASC and caspase-1, as well as HMGB1, were stained in control microglia and LPS-primed cells (Fig 1E).

In addition to the glial compartment, primary cortical neurons were analyzed by RT-PCR for the presence of inflammasome components and we could detect expression of *Nlrp1*, *Nlrp2*, *Nlrp6* and *Aim2*, whereas classical NLRP3 inflammasome components were only weakly expressed (Figure C in S1 File)

These results thus indicate that microglia are competent to form a functional NLRP3 inflammasome, whereas for astrocytes this type of inflammasome is probably not formed and its activation seems compromised.

### Microglia can secrete IL-1β and IL-18 in an NLRP3 inflammasome-dependent manner

**Fig 2:**
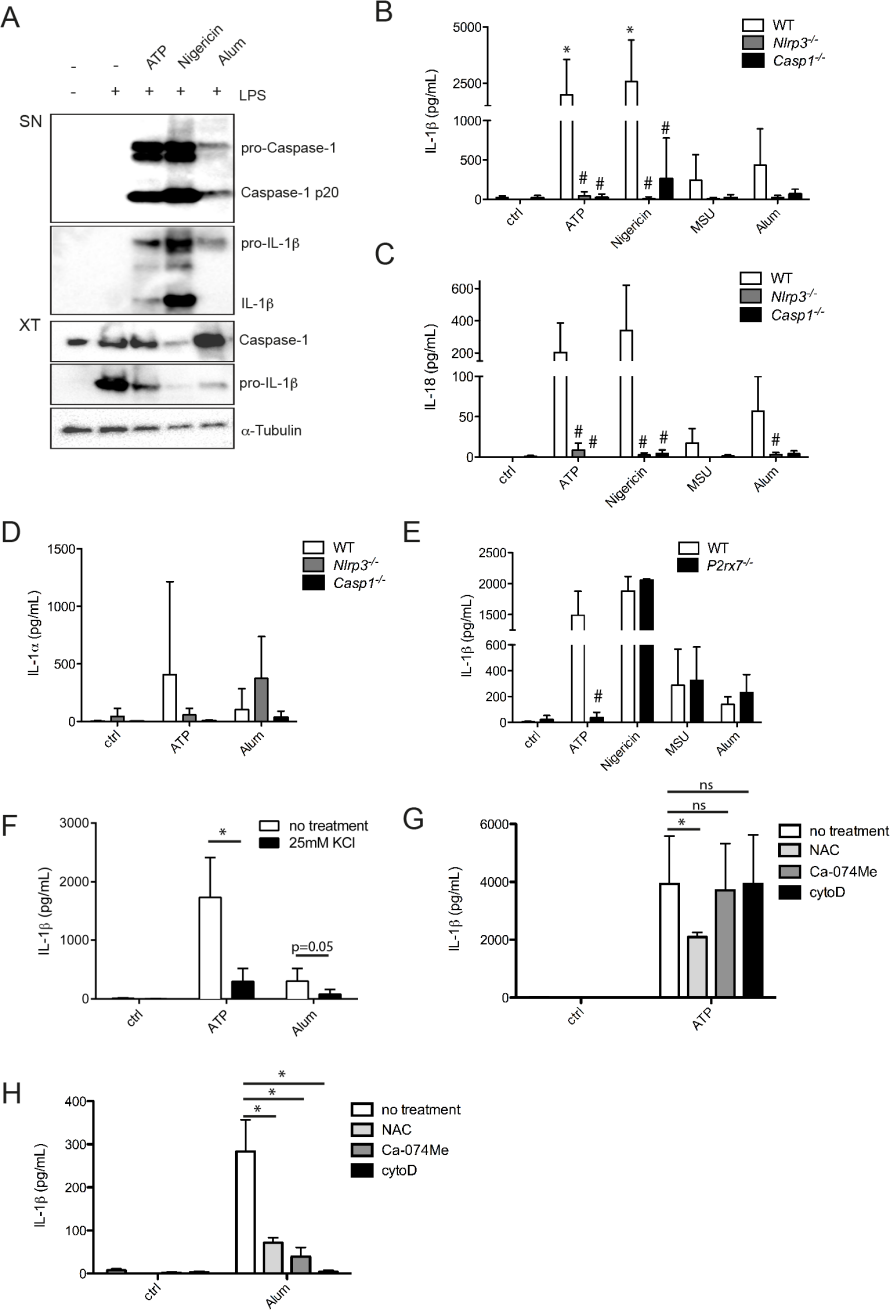
NLRP3 inflammasome activation mechanism in microglia. LPS primed microglia were stimulated with ATP (1 mM, 30 min), Nigericin (Nig, 1.34 μM, 2 h), Monosodium urate (MSU, 100 μg/ml, 5 h) or Aluminium hydroxide (Alum, 300 μg/ml, 5 h). IL-1β production and caspase-1 cleavage were assessed by Western blot (**A**). Secretion of IL-1β (**B**), IL-18 (**C**) and IL-1α(**D**) in supernatants of wild-type (WT), *Nlrp3* ^*-/-*^ and *Casp1* ^*-/-*^ microglia was assessed by ELISA. IL-1β secretion was assessed by ELISA in WT and *P2rx7* ^*-/-*^ microglia (**E**) and after treatment with different inhibitors (**F-H**) (± KCl (25 mM), N-acetyl cystein (NAC, 5 mM), Ca-074Me (10 μM) and cytochalasinD (cytoD, 2 μM)). Inhibitors were added for 30 min prior to inflammasome activation. Data shown are the mean ± SD of at least three independent experiments. *p<0.05, ns = not significant, compared to control (ctrl) (Fig 3B–3E) or compared to no treatment (Fig 3F–3H), #p<0.05, KO compared to WT.

As microglia are the brain resident macrophages and seem to express the minimal requirements for inflammasome activation, we investigated their ability to secrete IL-1β, IL-18 and IL-1α in response to classical inflammasome stimuli. When treated with widely used inflammasome activators, such as ATP and Nigericin, we observed robust IL-1β secretion and caspase-1 cleavage in LPS primed microglia (Fig 2A). In addition, particulate stimuli, such as monosodium urate (MSU) crystals or Alum, were also able to induce IL-1β secretion, although at lower levels (Fig 2A and 2B). Treatment with these different activators also resulted in IL-18 secretion by microglia (Fig 2C). Production of both IL-1β and IL-18 occurred in a NLRP3 inflammasome-dependent manner, as shown by the use of NLRP3- and caspase-1-deficient microglia, where the production of these cytokines was strongly reduced (Fig 2B and 2C). As both IL-1β and IL-1α are able to trigger the IL-1 receptor and their relative contributions *in vivo* are not well understood, we aimed to know if microglia could also produce IL-1α in response to inflammasome activation. We observed NLRP3 inflammasome-dependent production of IL-1α in response to ATP and Nigericin (Fig 2D and data not shown). In response to MSU and Alum, IL-1α was produced in a NLRP3- and caspase-1-independent manner, as also reported by others [31] (Fig 2D and data not shown). Production of all these cytokines required priming of microglia by LPS (Figure A in S2 File). Microglia also produced TNFα in response to these treatments, but this occurred in a NLRP3- and caspase-1-independent manner (Figure B in S2 File).

Inflammasome-dependent IL-1β production in microglia occurred through similar mechanisms than described in BMDMs. As expected, the P2X7 receptor was only required in response to stimulation with ATP, whereas Nigericin, MSU and Alum activated the inflammasome in a P2X7R-independent manner (Fig 2E). Potassium efflux was necessary for efficient inflammasome activation in microglia, as blocking it by addition of KCl to the extracellular medium strongly reduced IL-1β secretion (Fig 2F). This effect occurred in part at the priming step since we also observed reduced levels of pro-IL-1β (data not shown). Reactive oxygen species (ROS) were implicated in the microglial inflammasome activation process, since interfering with ROS production by N-acetyl cystein (NAC) impaired IL-1β secretion induced by ATP and Alum (Fig 2G and 2H). Lysosomal damage was implicated in inflammasome activation by Alum but not ATP, as shown by the use of the cathepsin B inhibitor Ca074-Me (Fig 2G and 2H). To determine if phagocytosis of Alum particles was required for inflammasome activation, we used the actin polymerization inhibitor cytochalasinD (cytoD). The presence of cytoD selectively reduced IL-1β secretion in response to Alum (Fig 2H), but not ATP (Fig 2G).

### Amyloid-β induced microglial inflammasome activation is P2X7R-independent

**Fig 3:**
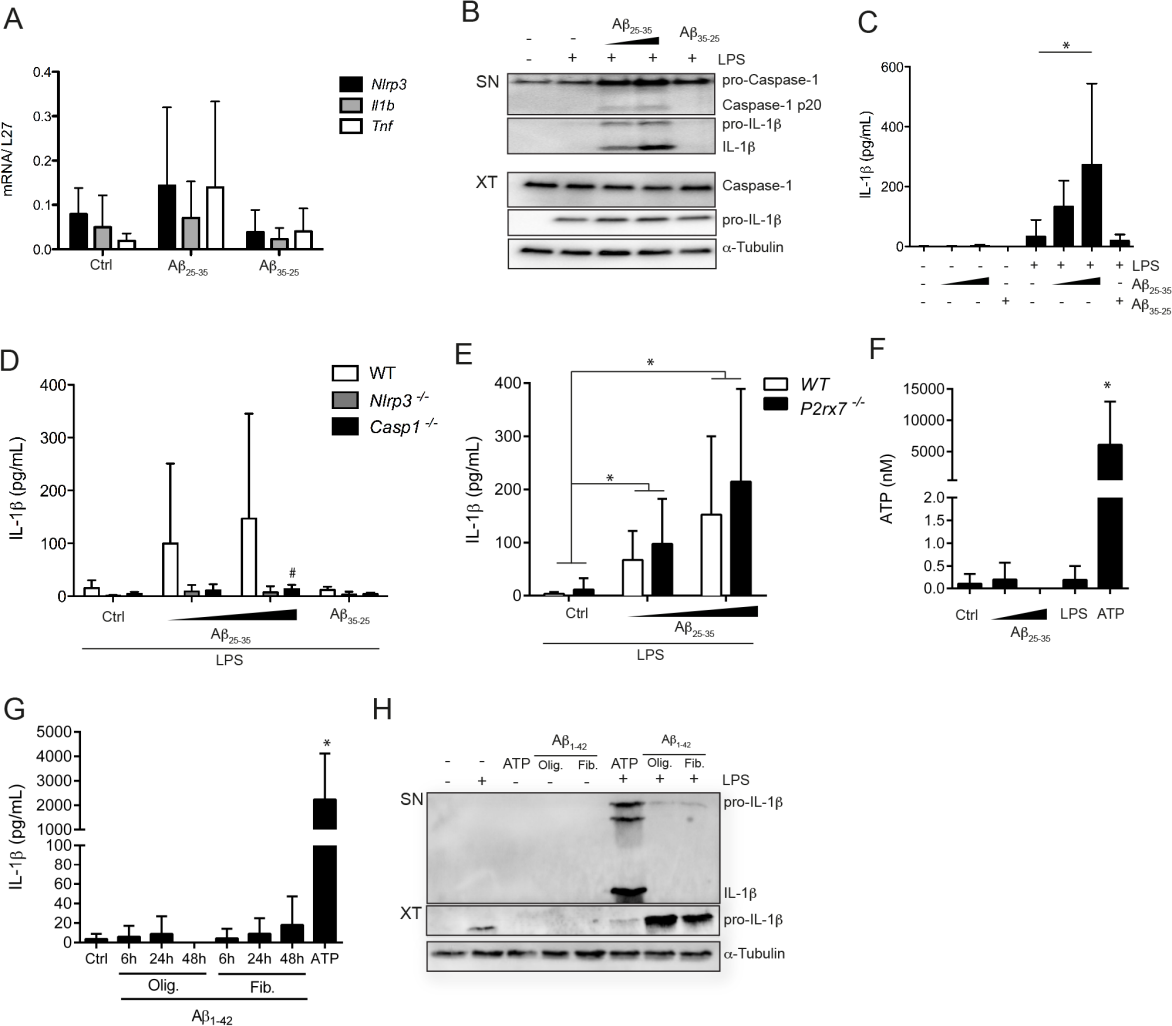
Amyloid-β_25–35_ activates microglia to secrete IL-1β independently from P2X7 signaling. Untreated or LPS primed microglia were stimulated with amyloid-β (Aβ)_**25–35**_ (20 or 50 μM) or Aβ_**35–25**_ (50 μM) for 5 h. (**A**) RNA was analyzed for expression of *Nlrp3*, *Il1b* and *Tnf*, relative to *L27*, by Real-Time PCR (**B)** Cell free culture supernatants (SN) and cell lysates (XT) were analyzed by Western Blot for expression of caspase-1 and IL-1β. α-Tubulin was used as loading control. (**C)** IL-1β production in culture supernatants was assessed by ELISA. (**D, E**) IL-1β production in culture supernatants was assessed by ELISA in wild type, *Nlrp3* ^*-/-*^ and *Casp1* ^*-/-*^ (**D**), or *P2rx7* ^*-/-*^ (**E**) microglia. (**F)** ATP release was quantified in cell supernatant upon treatment. (**G**) Untreated or LPS primed microglia were stimulated with amyloid-β (Aβ) 1–42 (10μM) for 6h, 24h or 48h. IL-1β production in culture supernatants was assessed by ELISA. (**H)** Cell free culture supernatants (SN) and cell lysates (XT) were analyzed by Western Blot for expression IL-1β. α-Tubulin was used as loading control (**I)**. Data shown are mean ± SD of at least three independent experiments. *p<0.05 compared to control (ctrl), #p<0.05, KO compared to WT.

Inflammasome function has been linked to a number of diseases that are characterized by peptide aggregates or particulate structures, such as gout or type 2 diabetes [32, 33]. Neurodegenerative conditions, such as Alzheimer’s or Parkinson’s disease, are known to involve plaques formed by amyloid aggregates during the course of the disease [34]. In order to better understand their potential role in the development of the disease and more specifically in microglial activation, we treated cells with the amyloid-β fragment Aβ_25–35_. This Aβ peptide is unable to prime microglia, as shown by the lack of upregulation of different genes, such as *Nlrp3*, *Il1b* or *Tnf* upon treatment (Fig 3A). However, Aβ_25–35_ is able to induce caspase-1 cleavage and IL-1β secretion in LPS primed microglia, whereas a control peptide (Aβ_35–25_) is not (Fig 3B and 3C). IL-1β production is NLRP3- and caspase-1-dependent, as shown by the strong reduction of the cytokine in inflammasome-deficient microglia (Fig 3D). However inflammasome activation by Aβ is P2X7R-independent (Fig 3E); there is no requirement for this receptor either directly or indirectly, as we could not detect ATP secretion in microglia treated with Aβ (Fig 3F). Opposed to published results we were unable to detect significant IL-1β production by microglia treated with the Aβ_1–42_ peptide whether we used oligomeric or fibrillar preparations of the peptide and different time points (Fig 3G and 3H) [18, 35, 36].

### α-synuclein can modestly prime microglia, but does not result in significant IL-1β secretion

**Fig 4:**
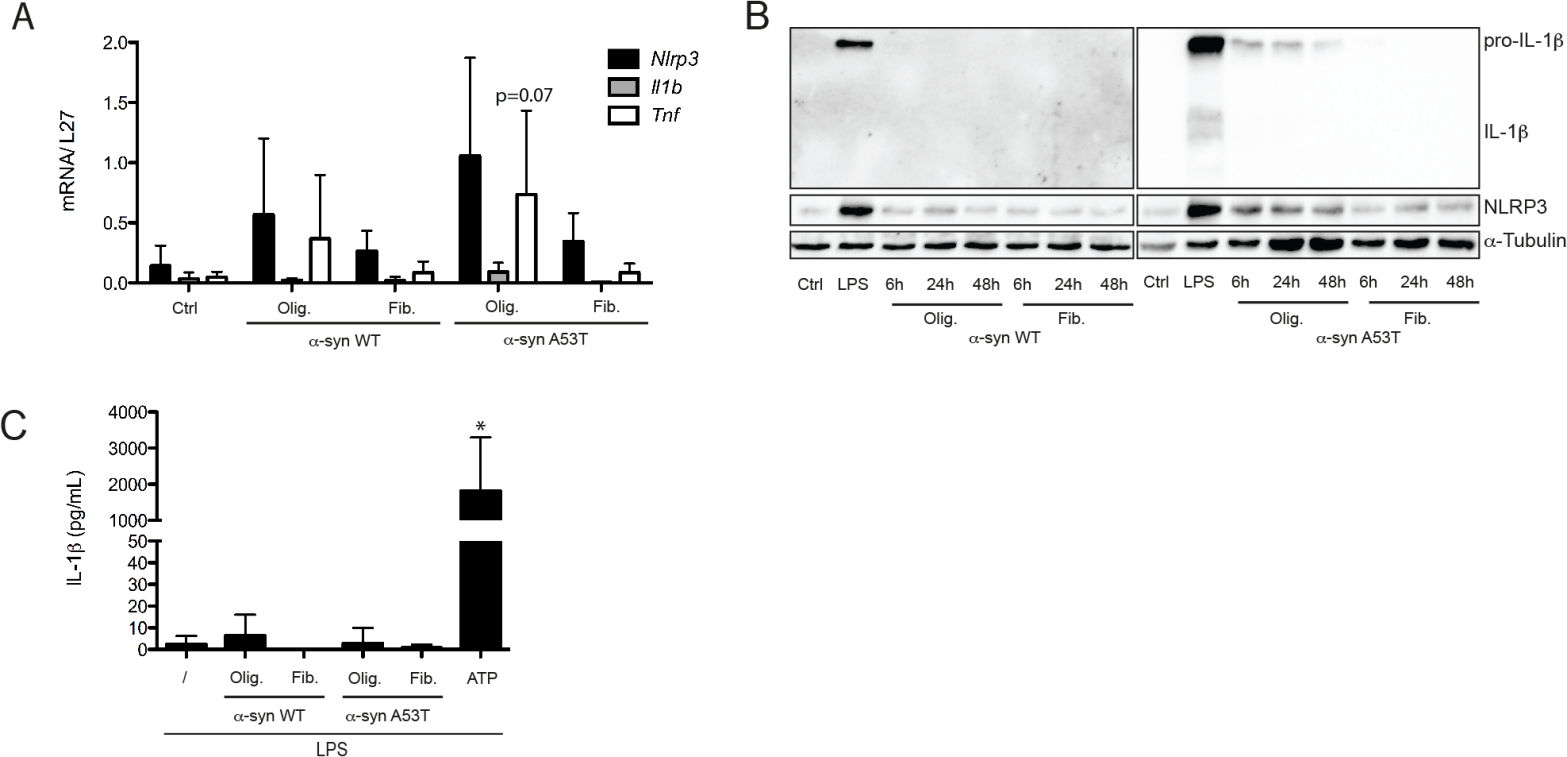
α-synuclein is not an NLRP3 inflammasome activator. Microglia were stimulated with 5 μM α-synuclein (α-syn), either wild-type (α-syn WT) or mutant (α-syn A53T) (oligomeric form: olig. or fibrillated form: fib.) for 6 h. (**A**) RNA was extracted and analyzed for expression of *Nlrp3*, *Il1b* and *Tnf*, relative to *L27*, by Real-Time PCR. (**B**) After 6 h, 24 h or 48 h of stimulation with α-syn, or 6 h with LPS, cell lysates were analyzed by Western Blot for expression of pro-IL-1β and NLRP3. α-Tubulin was used as loading control. (**C**) LPS primed microglia were stimulated for 6 h with α-syn or for 30 min with ATP (1 mM) and IL-1β production in culture supernatants was assessed by ELISA. Data shown are the mean ± SD of at least three independent experiments. *p<0.05 compared to control (ctrl).

In addition to amyloid-β, we focused on the ability of α-synuclein (α-syn) to trigger inflammasome activation. α-syn is a component of Lewy bodies that are formed during Parkinson’s disease and we were interested in its pro-inflammatory capacities as an aggregated peptide. Similarly to Aβ, α-syn can adopt different conformations such as oligomeric or fibrillar, and different degrees of toxicity are associated with each form. We therefore tested both forms for their ability to activate microglia. The oligomeric preparations showed slightly more transcriptional activation than the fibrillar forms concerning certain genes, such as *Tnf* or *Nlrp3* (Fig 4A). In addition to the native α-syn, we used a mutant peptide that occurs in familial forms of PD, the A53T mutant, and observed that this form has more pro-inflammatory potential than the WT form, but again only with the oligomeric conformation (Fig 4A). The same results were observed for pro-IL-1β and NLRP3 at protein level, when both α-syn WT and A53T forms (oligomeric and fibrillar) were used to treat microglia for longer time points (Fig 4B). When using α-syn preparations for inflammasome activation, we did not observe significant IL-1β secretion in LPS primed microglia in response to all tested preparations (Fig 4C).

### Astrocytes do not secrete IL-1β or IL-18 in response to inflammasome stimulation

As primary mixed glial cultures contain at least both glial cell types (astrocytes and microglia) and as microglia are extremely reactive immune cells, it is important to deplete them as much as possible from astrocyte cultures. Whereas microglia can be isolated to high purity by different techniques, astrocyte cultures mostly remain “contaminated” by microglia [37]. In order to analyze the purest possible astrocyte cultures *in vitro*, we performed double MACS isolation where astrocytes were negatively selected during the sorting [26]. In this way we were able to separate microglia and astrocytes efficiently starting from a mixed glial cell culture (MGC). We analyzed expression of different cell type specific markers (Iba1 and CD11b for microglia and GFAP for astrocytes) in the microglia fraction and in the astrocyte enriched culture-double MACS (AEC-M2), which were 98% and 99% pure respectively (Figures A and B in S1 File).

**Fig 5:**
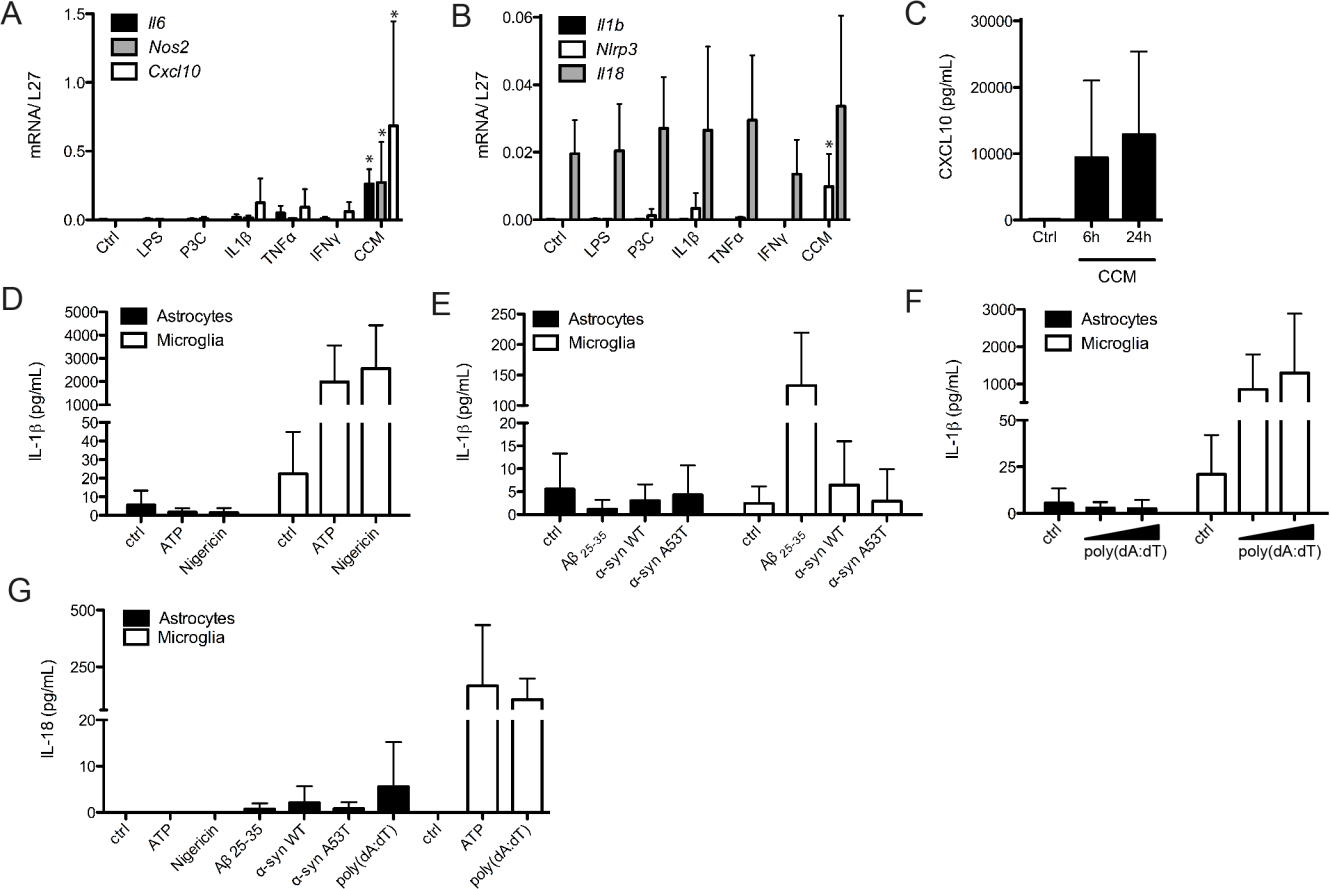
Astrocytes are not able to secrete inflammasome substrates IL-1β and IL-18. Astrocytes were treated for 6h with LPS, P3C, IL-1β, TNFα, IFNγ or CCM and RNA was extracted and analyzed for expression of *Il6*, *Nos2*, *Cxcl10* (**A**), *Il1b*, *Nlrp3 and IL18* **(B)** relative to *L27*, by Real-Time PCR. CXCL10 secretion was assessed after 6h or 24h of CCM treatment **(C)**. Mouse astrocyte-enriched cultures (AEC-M2) and microglia were primed with Complete Cytokine Mix for (24h) or LPS (6h), respectively, prior to stimulation with ATP (1 mM, 30 min), Nig (1,34 μM, 2h), Aβ_**25–35**_ (20 μM, 5h), WT and mutant α-synuclein (5 μM, 5h) or poly(dA:dT) (1 or 2,5 μg/mL, 5h). Levels of IL-1β(**D, E and F**) and IL-18 (**G**) in culture supernatants were assessed by ELISA. Data shown are the mean ± SD of at least three independent experiments *p<0.05 compared to control (ctrl).

In order to prime astrocytes for inflammasome activation we tested different products, such as LPS, P3C or different cytokines (IL-1β, TNFα and IFNγ) separately or in combination (CCM), and we observed that CCM was the most efficient in this cell type (Fig 5A). CCM treatment could induce transcriptional upregulation of *Nlrp3*, *Casp-1*, *Il6*, *Nos2* and *Cxcl10* (Fig 5A and 5B), as well as the secretion of CXCL10 in the supernatant (Fig 5C). We were however unable to detect substantial IL-1β or IL-18 secretion by CCM-primed astrocytes treated with the NLRP3 inflammasome activators ATP, Nigericin, Aβ or the alternative activator α-synuclein (Fig 5D, 5E and 5G). In addition, treatment with the AIM2 inflammasome activator poly(dA:dT) also failed to induce IL-1β or IL-18 secretion in astrocytes (Fig 5F and 5G). The substantial amounts of CXCL10 secreted by astrocytes in response to CCM indicate that these cells can play important roles in the brain immune response, but are not able themselves to secrete inflammasome-dependent cytokines, such as IL-1β or IL-18.

In order to differently address the question whether astrocytes are able to activate the classical NLRP3 inflammasome, we used neurosphere-derived astrocyte cultures [27]. When these cells were treated with LPS or CCM, we observed very low or no expression of *Asc* and *Il1b*, similarly to astrocyte-enriched cultures (Figure A in S2 File), suggesting again that astrocytes do not express a functional NLRP3 inflammasome under these conditions.

## Discussion

IL-1β production has been linked to neuroinflammatory conditions and neurodegenerative diseases for a long time with both beneficial and detrimental effects described. However the potential role of the NLRP3 inflammasome in the local production of IL-1β in the brain is not very well understood. Recently several reports started to investigate inflammasome activation in neuroinflammatory and neurodegenerative conditions, in spite of this fact a lot of basic questions remain. We therefore aimed to characterize in more detail the *in vitro* capacities of microglia and astrocytes separately to respond to inflammasome activation, since this issue has not clearly been addressed. Especially concerning astrocytes, it is not clear if they are able to express TLR4 and/or produce IL-1β as some conflicting reports exist [24, 38]. Analysis of this phenomenon has often been complicated by the fact that, according to purification protocols used, remaining microglia most probably "contaminate" astrocyte cultures. We observed that less than 5% remaining microglia could result in detectable IL-1β production and therefore we purified our astrocyte cultures by two successive MACS passages in order to carefully deplete microglia. Recently, Facci and coworkers demonstrated that rat microglia-free astrocytes are unable to secrete IL-1β, by depleting microglia with the lysosomotropic agent Leu-Leu-OMe, a completely different approach than our MACS purification [39]. We were unable to observe any substantial IL-1β and IL-18 secretion in astrocyte cultures in response to classical NLRP3 inflammasome stimuli. This could be due to the lack of ASC and weak NLRP3 expression in these cells. In addition we could not induce pro-IL-1β expression in astrocytes, although we tested several potential inducers, such as LPS, P3C or CCM, and longer time points. These observations were confirmed in neurosphere-derived astrocyte cultures that are completely devoid of microglia. It has also been shown that in human astrocytes IL-1β mRNA was not translated to protein, probably by an active repression mechanism [40].

Concerning the role of other NLR proteins in the CNS, there is one report describing NLRP2 inflammasome expression in human astrocytes [41]. We did however not detect NLRP2 expression in our mouse astrocyte cultures, suggesting species-specific differences in inflammasome components expression. Nlrp2 expression in the mouse seems to be restricted to ovaries and plays a role in early embryonic development as a maternal effect gene [42]. We were however able to detect NLRP1 expression in astrocytes, as also described before in several reports that link NLRP1 inflammasome activation in astrocytes and neurons to *in vivo* models of spinal cord injury and thromboembolic stroke [43, 44]. In addition to classical inflammasome activators, such as ATP or Nigericin, we also tested AIM2 activation by the use of poly(dA:dT) in astrocytes. In all cases, these potent microglial inflammasome activators did not result in detectable IL-1β or IL-18 production in astrocytes. As both NLRP3 and AIM2 are dependent on ASC in order to activate caspase-1, the lack of ASC in astrocytes could explain this observation. ASC-independent inflammasomes, such as NLRP1 or NLRC4 inflammasomes that can directly engage caspase-1 could lead to induction of pyroptosis and/or cleavage and secretion of IL-18. In addition the non-canonical inflammasome activation pathway could be functional in astrocytes, as we could show that caspase-11 expression can be upregulated upon priming.

A neuronal NLRP1 inflammasome has been described as a preassembled complex consisting of NLRP1, caspase-1, ASC, caspase-11 and XIAP [44]. We could demonstrate expression of ASC, caspase-1 and caspase-11 in cortical neurons, although they were weakly expressed. In contrast, NLRP1, NLRP2 and Aim2 were relatively abundant in cortical neurons. Functional analysis of these complexes has recently started and needs further characterization in order to better understand the importance of inflammasomes in neurons and neuronal disorders [45].

Microglia share several features of peripheral macrophages when they are isolated *in vitro*. They respond to classical NLRP3 inflammasome activation by secretion of IL-1β and other cytokines, such as IL-18, IL-1α and TNFα. IL-1β and IL-18 production in response to particulate stimuli, such as MSU or Alum, is weaker than after activation with ATP or Nigericin. We observed that microglial inflammasome activation occurs through similar mechanisms than in BMDMs. We could show that potassium efflux, ROS production and cathepsin B function are also required for efficient inflammasome activation in microglia.

A recent report showed that microglial IL-18 secretion in response to stimulation with *S*.*aureus* is NLRP3- and ASC-independent [46]. In microglia, activation by *S*.*aureus* seems to trigger additional pathways that can lead to mature IL-18, whereas in macrophages *S*.*aureus* induced IL-18 production has been described to be NLRP3 inflammasome-dependent [47]. We show that IL-18 production in microglia in response to classical activators, such as ATP, is NLRP3- and caspase-1-dependent as the corresponding knock-out microglia are no longer able to secrete IL-18. As IL-18 is emerging as an important player in neuroinflammation, this observation is clearly opening interesting questions concerning the differential production of IL-18 in the brain [13].

We were interested in the potential implication of the NLRP3 inflammasome in neurodegenerative diseases and focused on *in vitro* activation of microglia and astrocytes by peptide aggregates related to these conditions. Several reports already establish a link between amyloid-β and inflammasome-dependent IL-1β secretion in macrophages and microglia [18, 35, 36]. Despite published reports we were unsuccessful in triggering inflammasome activation by the full length peptide Aβ_1–42_. We tested fibrillar and oligomeric forms from different providers and were unable to detect IL-1β production in primary microglia. However, when we tested the peptide fragment Aβ_25–35_, we could observe inflammasome activation consistent with another report [35], as monitored by IL-1β secretion in microglia, but in a P2X7R-independent way (Fig 3E). The *in vivo* relevance of the NLRP3 inflammasome in Alzheimer's disease has recently been demonstrated in the APP/PS1 mouse model where NLRP3-deficient mice show less severe disease symptoms [19]. The conformation of Aβ peptides seems to be of great importance for immune activation *in vitro* and may explain the different results obtained [48]. In addition, several reports describe the ability of Aβ to directly prime microglia, resulting in proIL-1β production. We could not observe this with the Aβ _25–35_ peptide we used (Fig 3A); the Aβ _1–42_ may have more pro-inflammatory capacities. However, priming microglia or macrophages with LPS before activating the inflammasome with Aβ may be required for a robust activation and detection of IL-1β *in vitro*, as suggested by several published studies [18, 35, 36, 49, 50].

In correlation to Aβ, we tested the capacity of α-synuclein, a PD-related peptide aggregate, to activate the inflammasome in microglia. α-syn treatment did not result in mature IL-1β secretion in our hands, although we tested different kinetics and concentrations. Opposed to this are recent reports describing substantial triggering of the inflammasome by aggregated α-syn in human monocytes and THP1 cells [51, 52]. One explanation might be the different aggregation forms used in our study, in addition inflammasome activation in mouse macrophages and in human monocytes occurs through different mechanisms [53]. Astrocytes treated with Aβ_25–35_ or α-syn, did not secrete any IL-1β or IL-18 (Fig 5E) and only very modest levels of CXCL10 (data not shown), indicating that astrocytes do not strongly react to these peptides, at least *in vitro*. During the course of neurodegenerative diseases, it may be that microglial inflammasomes are preferentially activated in response to neuronal death and associated ATP release rather than by the presence of senile plaques and aggregated peptides. A recent study also underlines the fact that adult microglia considerably differ from neonatal microglia in their gene signature and this could also explain functional differences in inflammasome activation in the adult diseased brain [54].

It remains crucial to analyze microglia-astrocyte crosstalk *in vivo* to fully understand the importance of NLRP3 inflammasome function in the brain and the potential therapeutic interests in neurodegenerative diseases, yet our results suggest that microglia are the main cell type in the brain responsible for IL-1β and IL-18 secretion.

## Supporting Information

S1 File Characterization of cell expression markers and inflammasome components in primary microglia, astrocytes and neurosphere-derived astrocytes. (Figure A) mRNA expression of *Aif1*, *Itgam*, *Tlr4* (microglia markers) and *Gfap* (astrocyte marker) has been analyzed in mixed glial cultures (MGC P0), in astrocyte-enriched cultures (AEC) after one (AEC-M1) or two MACS purifications (AEC-M2) and in microglial cultures, relative to L27, by Real-Time PCR. (Figure B) MGC P0, microglia and AEC-M2 were analyzed by immunofluorescence for the expression of *Iba1* and *GFAP*, nuclei are stained with DAPI. Data shown are representative of at least three independent experiments. (Figure C) mRNA expression of inflammasome components, substrates or related genes has been analyzed in microglia, astrocytes (AEC-M2), BMDM and cortical neurons, after 6 h of stimulation with LPS for microglia and BMDM, Complete Cytokine Mix for astrocytes, or in unstimulated neurons. Data in the table are given as means of Ct ± SD. Data shown are representative of at least three independent experiments.
(TIF)

S2 File Priming is required for inflammasome activation in microglia (Figure A) IL-1β, IL-18 and IL-1α production in culture supernatants was assessed by ELISA in microglia after stimulation with ATP (1 mM), Nigericin (Nig, 1.34 μM), Monosodium Urate (MSU), 100 μg/ml) or Aluminium hydroxide (Alum, 300 μg/ml), in cells either primed with LPS or not. Data are means ± SD values of triplicate wells. (Figure B) TNF-α production in culture supernatants was assessed by ELISA in microglia of wild-type, Nlrp3^*-/-*^ or Casp1^*-/-*^ mice. Data are means ± SD values of triplicate wells. Data shown are representative of at least three independent experiments. (Figure C) mRNA expression of *Nlrp3*, *Pycard*, *casp1*, *Il1b* and *Il18* has been analyzed in neurosphere-derived astrocyte cultures stimulated for 6h with LPS or CCM, relative to L27, by Real-Time PCR. Data are means ± SD values of at least three independent experiments.
(TIF)

## References

[1] Allan SM, Rothwell NJ. Cytokines and acute neurodegeneration. Nat Rev Neurosci. 2001;2(10):734–44. Epub 2001/10/05. 10.1038/35094583 35094583 [pii]. . 11584311

[2] Shie FS, Woltjer RL. Manipulation of microglial activation as a therapeutic strategy in Alzheimer's disease. Curr Med Chem. 2007;14(27):2865–71. Epub 2007/11/30. . 18045132 10.2174/092986707782359981

[3] Koprich JB, Reske-Nielsen C, Mithal P, Isacson O. Neuroinflammation mediated by IL-1beta increases susceptibility of dopamine neurons to degeneration in an animal model of Parkinson's disease. J Neuroinflammation. 2008;5:8. Epub 2008/02/29. doi: 1742-2094-5-8 [pii] 10.1186/1742-2094-5-8 18304357 PMC2292163

[4] Glass CK, Saijo K, Winner B, Marchetto MC, Gage FH. Mechanisms underlying inflammation in neurodegeneration. Cell. 2010;140(6):918–34. 10.1016/j.cell.2010.02.016 20303880 PMC2873093

[5] Allan SM, Tyrrell PJ, Rothwell NJ. Interleukin-1 and neuronal injury. Nat Rev Immunol. 2005;5(8):629–40. Epub 2005/07/22. doi: nri1664 [pii] 10.1038/nri1664 . 16034365

[6] Simi A, Tsakiri N, Wang P, Rothwell NJ. Interleukin-1 and inflammatory neurodegeneration. Biochem Soc Trans. 2007;35(Pt 5):1122–6. Epub 2007/10/25. doi: BST0351122 [pii] 10.1042/BST0351122 . 17956293

[7] Steinman L. Inflammatory cytokines at the summits of pathological signal cascades in brain diseases. Sci Signal. 2013;6(258):pe3. Epub 2013/01/17. doi: scisignal.2003898 [pii] 10.1126/scisignal.2003898 . 23322904

[8] Martinon F, Burns K, Tschopp J. The inflammasome: a molecular platform triggering activation of inflammatory caspases and processing of proIL-beta. Mol Cell. 2002;10(2):417–26. . 12191486 10.1016/s1097-2765(02)00599-3

[9] Latz E, Xiao TS, Stutz A. Activation and regulation of the inflammasomes. Nat Rev Immunol. 2013;13(6):397–411. Epub 2013/05/25. doi: nri3452 [pii] 10.1038/nri3452 . 23702978 PMC3807999

[10] Walsh JG, Muruve DA, Power C. Inflammasomes in the CNS. Nat Rev Neurosci. 2014;15(2):84–97. Epub 2014/01/09. doi: nrn3638 [pii] 10.1038/nrn3638 . 24399084

[11] Lamkanfi M, Sarkar A, Vande Walle L, Vitari AC, Amer AO, Wewers MD, et al Inflammasome-dependent release of the alarmin HMGB1 in endotoxemia. J Immunol. 2010;185(7):4385–92. Epub 2010/08/31. doi: jimmunol.1000803 [pii] 10.4049/jimmunol.1000803 20802146 PMC3428148

[12] Lu B, Nakamura T, Inouye K, Li J, Tang Y, Lundback P, et al Novel role of PKR in inflammasome activation and HMGB1 release. Nature. 2012;488(7413):670–4. Epub 2012/07/18. doi: nature11290 [pii] 10.1038/nature11290 . 22801494 PMC4163918

[13] Felderhoff-Mueser U, Schmidt OI, Oberholzer A, Buhrer C, Stahel PF. IL-18: a key player in neuroinflammation and neurodegeneration? Trends Neurosci. 2005;28(9):487–93. Epub 2005/07/19. doi: S0166-2236(05)00166-9 [pii] 10.1016/j.tins.2005.06.008 . 16023742

[14] Moynagh PN. The interleukin-1 signalling pathway in astrocytes: a key contributor to inflammation in the brain. J Anat. 2005;207(3):265–9. Epub 2005/09/28. doi: JOA445 [pii] 10.1111/j.1469-7580.2005.00445.x 16185251 PMC1571539

[15] Ratsimandresy RA, Dorfleutner A, Stehlik C. An Update on PYRIN Domain-Containing Pattern Recognition Receptors: From Immunity to Pathology. Front Immunol. 2013;4:440 Epub 2013/12/25. 10.3389/fimmu.2013.00440 24367371 PMC3856626

[16] Wen H, Miao EA, Ting JP. Mechanisms of NOD-like receptor-associated inflammasome activation. Immunity. 2013;39(3):432–41. Epub 2013/09/24. doi: S1074-7613(13)00389-0 [pii] 10.1016/j.immuni.2013.08.037 24054327 PMC3835203

[17] Inoue M, Shinohara ML. NLRP3 Inflammasome and MS/EAE. Autoimmune Dis. 2012;2013:859145 Epub 2013/02/01. 10.1155/2013/859145 23365725 PMC3556409

[18] Halle A, Hornung V, Petzold GC, Stewart CR, Monks BG, Reinheckel T, et al The NALP3 inflammasome is involved in the innate immune response to amyloid-beta. Nat Immunol. 2008;9(8):857–65. Epub 2008/07/08. doi: ni.1636 [pii] 10.1038/ni.1636 . 18604209 PMC3101478

[19] Heneka MT, Kummer MP, Stutz A, Delekate A, Schwartz S, Vieira-Saecker A, et al NLRP3 is activated in Alzheimer's disease and contributes to pathology in APP/PS1 mice. Nature. 2013;493(7434):674–8. Epub 2012/12/21. doi: nature11729 [pii] 10.1038/nature11729 . 23254930 PMC3812809

[20] Hafner-Bratkovic I, Bencina M, Fitzgerald KA, Golenbock D, Jerala R. NLRP3 inflammasome activation in macrophage cell lines by prion protein fibrils as the source of IL-1beta and neuronal toxicity. Cell Mol Life Sci. 2012;69(24):4215–28. Epub 2012/08/29. 10.1007/s00018-012-1140-0 22926439 PMC3508391

[21] Shi F, Yang L, Kouadir M, Yang Y, Wang J, Zhou X, et al The NALP3 inflammasome is involved in neurotoxic prion peptide-induced microglial activation. J Neuroinflammation. 2012;9:73. Epub 2012/04/26. doi: 1742-2094-9-73 [pii] 10.1186/1742-2094-9-73 22531291 PMC3394218

[22] Masters SL, Dunne A, Subramanian SL, Hull RL, Tannahill GM, Sharp FA, et al Activation of the NLRP3 inflammasome by islet amyloid polypeptide provides a mechanism for enhanced IL-1beta in type 2 diabetes. Nat Immunol. 2010;11(10):897–904. Epub 2010/09/14. doi: ni.1935 [pii] 10.1038/ni.1935 20835230 PMC3103663

[23] Sofroniew MV, Vinters HV. Astrocytes: biology and pathology. Acta Neuropathol. 2010;119(1):7–35. Epub 2009/12/17. 10.1007/s00401-009-0619-8 20012068 PMC2799634

[24] Hanamsagar R, Hanke ML, Kielian T. Toll-like receptor (TLR) and inflammasome actions in the central nervous system. Trends Immunol. 2012;33(7):333–42. Epub 2012/04/24. doi: S1471-4906(12)00051-8 [pii] 10.1016/j.it.2012.03.001 22521509 PMC3383346

[25] Martinon F, Petrilli V, Mayor A, Tardivel A, Tschopp J. Gout-associated uric acid crystals activate the NALP3 inflammasome. Nature. 2006;440(7081):237–41. . 16407889 10.1038/nature04516

[26] Losciuto S, Dorban G, Gabel S, Gustin A, Hoenen C, Grandbarbe L, et al An efficient method to limit microglia-dependent effects in astroglial cultures. J Neurosci Methods. 2012;207(1):59–71. Epub 2012/04/10. doi: S0165-0270(12)00105-7 [pii] 10.1016/j.jneumeth.2012.03.010 . 22483759

[27] Crocker SJ, Frausto RF, Whitton JL, Milner R. A novel method to establish microglia-free astrocyte cultures: comparison of matrix metalloproteinase expression profiles in pure cultures of astrocytes and microglia. Glia. 2008;56(11):1187–98. Epub 2008/05/02. 10.1002/glia.20689 18449943 PMC2776034

[28] Guarda G, Dostert C, Staehli F, Cabalzar K, Castillo R, Tardivel A, et al T cells dampen innate immune responses through inhibition of NLRP1 and NLRP3 inflammasomes. Nature. 2009;460(7252):269–73. Epub 2009/06/06. doi: nature08100 [pii] 10.1038/nature08100 . 19494813

[29] Gonthier B, Koncina E, Satkauskas S, Perraut M, Roussel G, Aunis D, et al A PKC-dependent recruitment of MMP-2 controls semaphorin-3A growth-promoting effect in cortical dendrites. PLoS One. 2009;4(4):e5099 Epub 2009/04/09. 10.1371/journal.pone.0005099 19352510 PMC2663036

[30] Henn A, Kirner S, Leist M. TLR2 hypersensitivity of astrocytes as functional consequence of previous inflammatory episodes. J Immunol. 2011;186(5):3237–47. Epub 2011/02/02. doi: jimmunol.1002787 [pii] 10.4049/jimmunol.1002787 . 21282508

[31] Gross O, Yazdi AS, Thomas CJ, Masin M, Heinz LX, Guarda G, et al Inflammasome activators induce interleukin-1alpha secretion via distinct pathways with differential requirement for the protease function of caspase-1. Immunity. 2012;36(3):388–400. Epub 2012/03/27. doi: S1074-7613(12)00093-3 [pii] 10.1016/j.immuni.2012.01.018 . 22444631

[32] Masters SL O 'Neill LA. Disease-associated amyloid and misfolded protein aggregates activate the inflammasome. Trends Mol Med. 2011;17(5):276–82. Epub 2011/03/08. doi: S1471-4914(11)00006-2 [pii] 10.1016/j.molmed.2011.01.005 . 21376667

[33] Robbins GR, Wen H, Ting JP. Inflammasomes and metabolic disorders: old genes in modern diseases. Mol Cell. 2014;54(2):297–308. Epub 2014/04/29. doi: S1097-2765(14)00262-7 [pii] 10.1016/j.molcel.2014.03.029 24766894 PMC4084585

[34] Heneka MT, Kummer MP, Latz E. Innate immune activation in neurodegenerative disease. Nat Rev Immunol. 2014;14(7):463–77. Epub 2014/06/26. doi: nri3705 [pii] 10.1038/nri3705 . 24962261

[35] Sanz JM, Chiozzi P, Ferrari D, Colaianna M, Idzko M, Falzoni S, et al Activation of microglia by amyloid {beta} requires P2X7 receptor expression. J Immunol. 2009;182(7):4378–85. Epub 2009/03/21. doi: 182/7/4378 [pii] 10.4049/jimmunol.0803612 . 19299738

[36] Parajuli B, Sonobe Y, Horiuchi H, Takeuchi H, Mizuno T, Suzumura A. Oligomeric amyloid beta induces IL-1beta processing via production of ROS: implication in Alzheimer's disease. Cell Death Dis. 2013;4:e975. Epub 2013/12/21. doi: cddis2013503 [pii] 10.1038/cddis.2013.503 24357806 PMC3877570

[37] Saura J. Microglial cells in astroglial cultures: a cautionary note. J Neuroinflammation. 2007;4:26. Epub 2007/10/17. doi: 1742-2094-4-26 [pii] 10.1186/1742-2094-4-26 17937799 PMC2140055

[38] Farina C, Aloisi F, Meinl E. Astrocytes are active players in cerebral innate immunity. Trends Immunol. 2007;28(3):138–45. Epub 2007/02/06. doi: S1471-4906(07)00024-5 [pii] 10.1016/j.it.2007.01.005 . 17276138

[39] Facci L, Barbierato M, Marinelli C, Argentini C, Skaper SD, Giusti P. Toll-like receptors 2, -3 and -4 prime microglia but not astrocytes across central nervous system regions for ATP-dependent interleukin-1beta release. Sci Rep. 2014;4:6824. Epub 2014/10/30. doi: srep06824 [pii] 10.1038/srep06824 . 25351234 PMC5381369

[40] Tarassishin L, Casper D, Lee SC. Aberrant expression of interleukin-1beta and inflammasome activation in human malignant gliomas. PLoS One. 2014;9(7):e103432 Epub 2014/07/24. 10.1371/journal.pone.0103432 PONE-D-14-17909 [pii]. 25054228 PMC4108401

[41] Minkiewicz J, de Rivero Vaccari JP, Keane RW. Human astrocytes express a novel NLRP2 inflammasome. Glia. 2013;61(7):1113–21. Epub 2013/04/30. 10.1002/glia.22499 . 23625868

[42] Peng H, Chang B, Lu C, Su J, Wu Y, Lv P, et al Nlrp2, a maternal effect gene required for early embryonic development in the mouse. PLoS One. 2012;7(1):e30344 Epub 2012/02/02. 10.1371/journal.pone.0030344 PONE-D-11-21105 [pii]. 22295082 PMC3266252

[43] Abulafia DP, de Rivero Vaccari JP, Lozano JD, Lotocki G, Keane RW, Dietrich WD. Inhibition of the inflammasome complex reduces the inflammatory response after thromboembolic stroke in mice. J Cereb Blood Flow Metab. 2009;29(3):534–44. Epub 2008/12/11. doi: jcbfm2008143 [pii] 10.1038/jcbfm.2008.143 . 19066616

[44] de Rivero Vaccari JP, Lotocki G, Marcillo AE, Dietrich WD, Keane RW. A molecular platform in neurons regulates inflammation after spinal cord injury. J Neurosci. 2008;28(13):3404–14. Epub 2008/03/28. doi: 28/13/3404 [pii] 10.1523/JNEUROSCI.0157-08.2008 . 18367607 PMC6670583

[45] de Rivero Vaccari JP, Dietrich WD, Keane RW. Activation and regulation of cellular inflammasomes: gaps in our knowledge for central nervous system injury. J Cereb Blood Flow Metab. 2014;34(3):369–75. Epub 2014/01/09. doi: jcbfm2013227 [pii] 10.1038/jcbfm.2013.227 24398940 PMC3948131

[46] Hanamsagar R, Torres V, Kielian T. Inflammasome activation and IL-1beta/IL-18 processing are influenced by distinct pathways in microglia. J Neurochem. 2011;119(4):736–48. Epub 2011/09/15. 10.1111/j.1471-4159.2011.07481.x 21913925 PMC3202981

[47] Mariathasan S, Weiss DS, Newton K, McBride J, O'Rourke K, Roose-Girma M, et al Cryopyrin activates the inflammasome in response to toxins and ATP. Nature. 2006;440(7081):228–32. . 16407890 10.1038/nature04515

[48] Paranjape GS, Gouwens LK, Osborn DC, Nichols MR. Isolated amyloid-beta(1–42) protofibrils, but not isolated fibrils, are robust stimulators of microglia. ACS Chem Neurosci. 2012;3(4):302–11. Epub 2012/08/04. 10.1021/cn2001238 22860196 PMC3402375

[49] El Khoury JB, Moore KJ, Means TK, Leung J, Terada K, Toft M, et al CD36 mediates the innate host response to beta-amyloid. J Exp Med. 2003;197(12):1657–66. Epub 2003/06/11. 10.1084/jem.20021546 jem.20021546 [pii]. 12796468 PMC2193948

[50] Sheedy FJ, Grebe A, Rayner KJ, Kalantari P, Ramkhelawon B, Carpenter SB, et al CD36 coordinates NLRP3 inflammasome activation by facilitating intracellular nucleation of soluble ligands into particulate ligands in sterile inflammation. Nat Immunol. 2013;14(8):812–20. Epub 2013/07/03. doi: ni.2639 [pii] 10.1038/ni.2639 23812099 PMC3720827

[51] Codolo G, Plotegher N, Pozzobon T, Brucale M, Tessari I, Bubacco L, et al Triggering of inflammasome by aggregated alpha-synuclein, an inflammatory response in synucleinopathies. PLoS One. 2013;8(1):e55375 Epub 2013/02/06. 10.1371/journal.pone.0055375 PONE-D-12-28132 [pii]. 23383169 PMC3561263

[52] Freeman D, Cedillos R, Choyke S, Lukic Z, McGuire K, Marvin S, et al Alpha-synuclein induces lysosomal rupture and cathepsin dependent reactive oxygen species following endocytosis. PLoS One. 2013;8(4):e62143 Epub 2013/05/02. 10.1371/journal.pone.0062143 PONE-D-12-39385 [pii]. 23634225 PMC3636263

[53] Rubartelli A, Gattorno M, Netea MG, Dinarello CA. Interplay between redox status and inflammasome activation. Trends Immunol. 2011;32(12):559–66. Epub 2011/10/04. doi: S1471-4906(11)00146-3 [pii] 10.1016/j.it.2011.08.005 . 21962746

[54] Butovsky O, Jedrychowski MP, Moore CS, Cialic R, Lanser AJ, Gabriely G, et al Identification of a unique TGF-beta-dependent molecular and functional signature in microglia. Nat Neurosci. 2014;17(1):131–43. Epub 2013/12/10. doi: nn.3599 [pii] 10.1038/nn.3599 24316888 PMC4066672

