# Supplementary figures and images for: NLRP3 Inflammasome Is Expressed and Functional in Mouse Brain Microglia but Not in Astrocytes
Source: PLoS One. 2015 Jun 19;10(6):e0130624. doi: 10.1371/journal.pone.0130624 (PMC4474809; doi:10.1371/journal.pone.0130624)

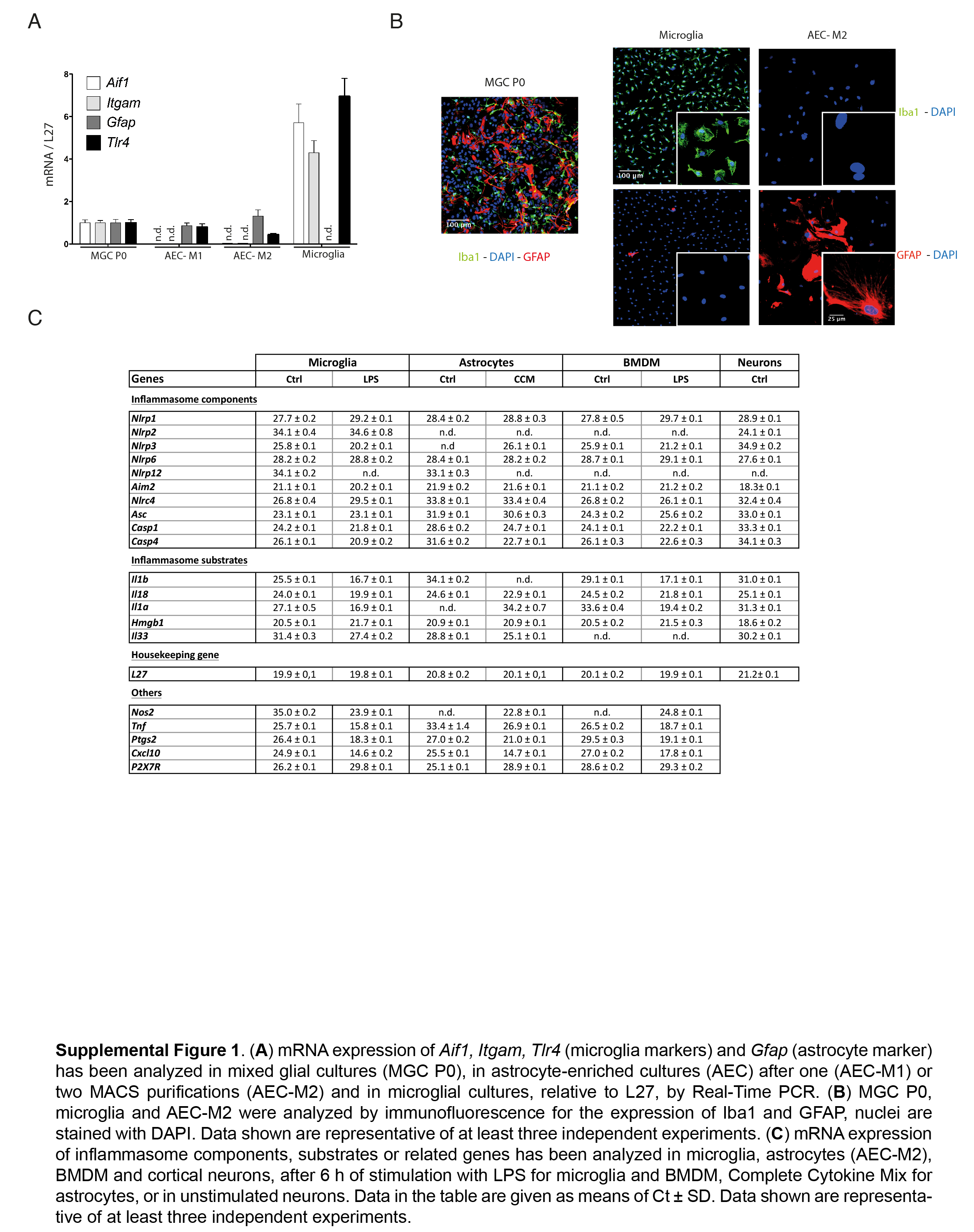

Supplement: S1 File — (Figure A) mRNA expression of Aif1, Itgam, Tlr4 (microglia markers) and Gfap (astrocyte marker) has been analyzed in mixed glial cultures (MGC P0), in astrocyte-enriched cultures (AEC) after one (AEC-M1) or two MACS purifications (AEC-M2) and in microglial cultures, relative to L27, by Real-Time PCR. (Figure B) MGC P0, microglia and AEC-M2 were analyzed by immunofluorescence for the expression of Iba1 and GFAP, nuclei are stained with DAPI. Data shown are representative of at least three independent experiments. (Figure C) mRNA expression of inflammasome components, substrates or related genes has been analyzed in microglia, astrocytes (AEC-M2), BMDM and cortical neurons, after 6 h of stimulation with LPS for microglia and BMDM, Complete Cytokine Mix for astrocytes, or in unstimulated neurons. Data in the table are given as means of Ct ± SD. Data shown are representative of at least three independent experiments. (TIF) [file pone.0130624.s001.tif]

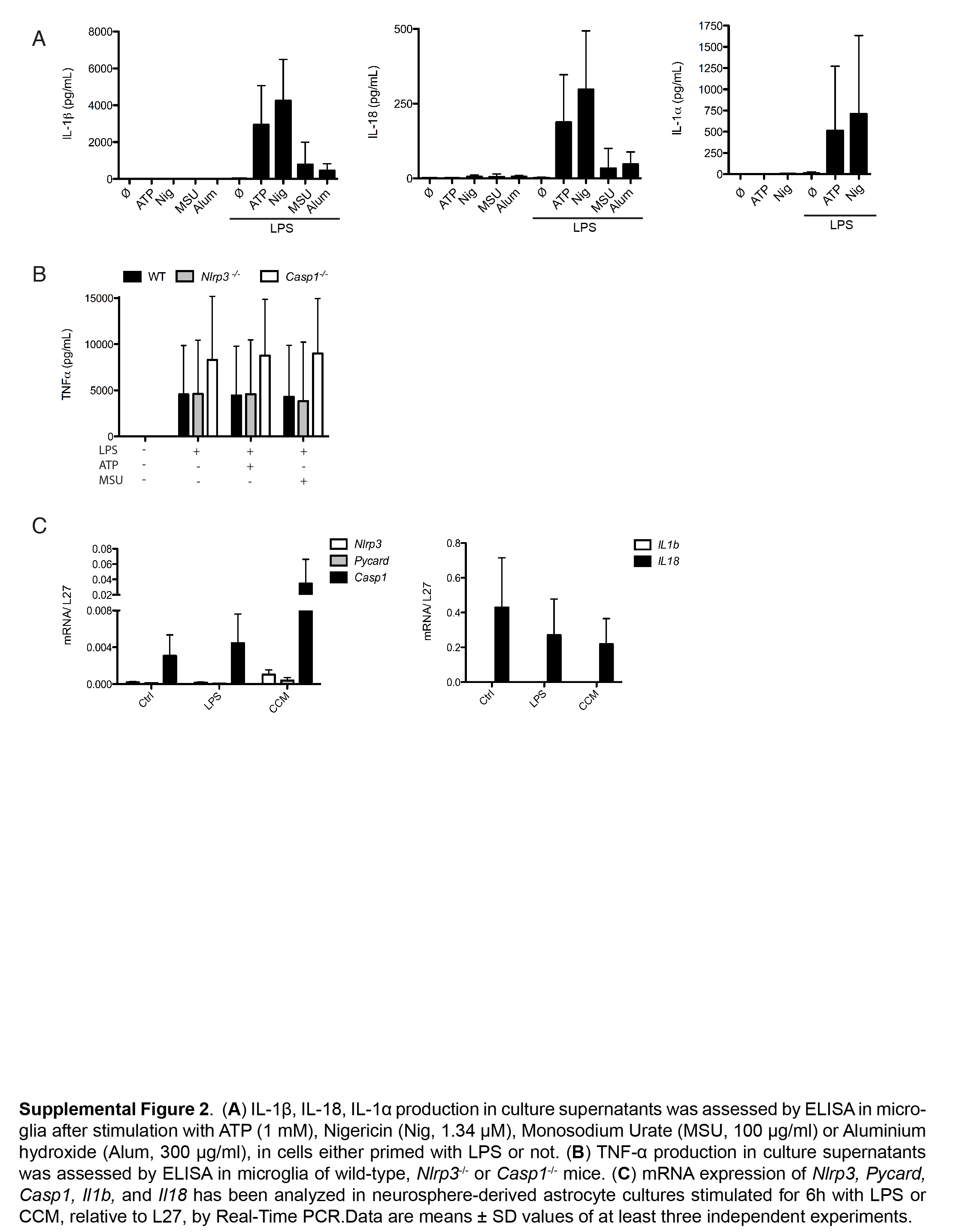

Supplement: S2 File — Data are means ± SD values of triplicate wells. (Figure B) TNF-α production in culture supernatants was assessed by ELISA in microglia of wild-type, Nlrp3-/- or Casp1-/- mice. Data are means ± SD values of triplicate wells. Data shown are representative of at least three independent experiments. (Figure C) mRNA expression of Nlrp3, Pycard, casp1, Il1b and Il18 has been analyzed in neurosphere-derived astrocyte cultures stimulated for 6h with LPS or CCM, relative to L27, by Real-Time PCR. Data are means ± SD values of at least three independent experiments. (TIF) [file pone.0130624.s002.tif]
